# Diabetic kidney disease in rural Australia: prevention, management, treatment and way forward

**DOI:** 10.3389/fmed.2025.1561566

**Published:** 2025-06-12

**Authors:** Allen G. Ross, Utpal K. Mondal, Anayochukwu E. Anyasodor, Shakeel Mahmood, Feleke H. Astawesegn, M. Mamun Huda, Subash Thapa, Setognal B. Aychiluhm, Santosh Giri, Md. Ferdous Rahman, Muhammad J. A. Shiddiky, Mohammad A. Moni, Kedir Y. Ahmed

**Affiliations:** Rural Health Research Institute, Charles Sturt University, Orange, NSW, Australia

**Keywords:** diabetic kidney disease (DKD), chronic kidney disease (CKD), type 2 diabetes, prevention, management, rural Australia

## Abstract

Diabetic kidney disease is a significant microvascular complication associated with chronic diabetes, contributing substantially to the overall health burden of the disease. This perspective focusses on evaluating the most recent advancements in screening techniques, prevention, and treatment strategies along with new advances in the field. A comprehensive literature search was conducted across PubMed, Scopus and Google Scholar databases to identify and synthesize recent evidence. In Australia, chronic kidney disease (CKD) was responsible for approximately two million hospital admissions, accounting for 18% of all hospitalizations in 2021–22. In remote areas, 17,100 CKD-related hospitalizations were reported during this period, with residents being three times more likely to be hospitalized for CKD compared to those living in major cities. Among First Nations people, the burden was 7.8 times higher than that of non-Indigenous populations. Advocacy for policy changes to address healthcare disparities in rural and remote Australia is crucial.

## Introduction

**Figure 1:**
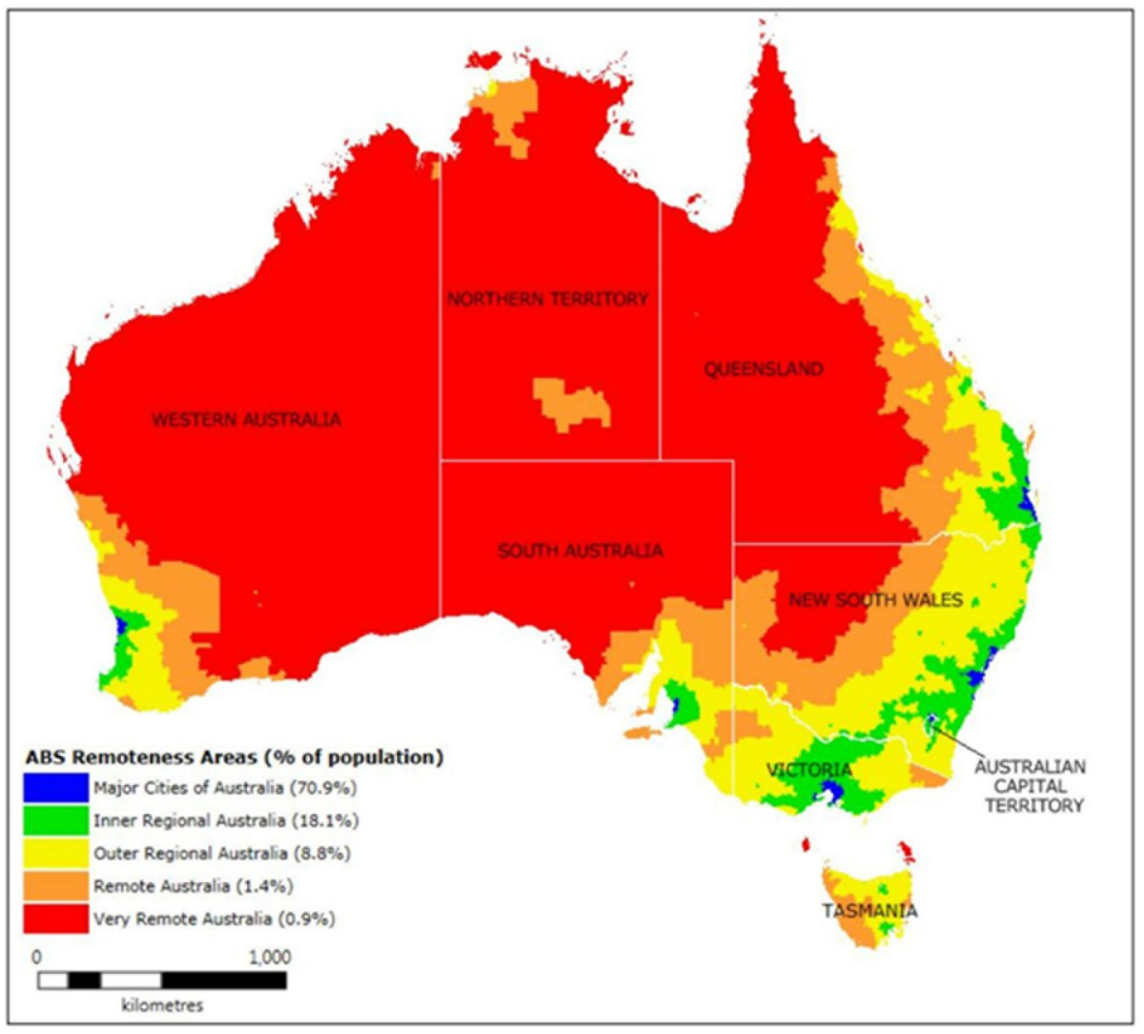
Distribution of the Australian population by remoteness areas as defined by the Australian Bureau of Statistics (ABS).

Diabetic kidney disease (DKD) is a leading cause of chronic kidney disease (CKD) and end-stage kidney disease (ESKD) globally, accounting for approximately 40–50% of all ESKD cases in high-income countries (1, 2). Despite advances in screening and treatment, disparities in access to early diagnosis and optimal care remain evident across healthcare systems. In Australia, the healthcare system provides universal access through Medicare, including subsidized medications via the Pharmaceutical Benefits Scheme (PBS) (3). However, rural and remote populations continue to face persistent barriers, including fewer healthcare facilities, limited access to specialists, and higher out-of-pocket costs (4, 5). Australia’s population is unevenly distributed, with approximately 30% of people residing outside major cities in rural and remote areas (4) (Figure 1). These population face unique challenges related to geographic isolation, including limited access to healthcare services, widespread workforce shortages, and less developed health infrastructure, all of which contribute to poorer health outcomes compared to those living in metropolitan regions (4). These inequities are further compounded for First Nations Australians, who experience a disproportionately high burden of CKD due to intersecting social, economic, and systemic barriers, including remoteness, racism, and lack of culturally safe care (6, 7). Notably, First Nations Australians are almost three times more likely to be living with diabetes compared to non-Indigenous Australians, placing them at significantly greater risk of developing DKD and related complications (8). Although some remote Australian communities lie only a few hundred kilometers from major metropolitan centers, they often face limited access to essential healthcare due to sparse infrastructure and chronic workforce shortages (4).

DKD is one of several microvascular complications associated with chronic diabetes, alongside diabetic retinopathy, neuropathy [including peripheral, autonomic, gastrointestinal, and genitourinary form (i.e., erectile dysfunction), neuropathic pain, orthostatic hypotension] and diabetic foot syndrome. These complications are frequently linked to poor glycaemic control, hypertension, and dyslipidaemia in both type 1 and type 2 diabetes (1, 2, 9, 10). Individuals diagnosed with diabetes at a younger age (<60 years) require intensive management and tight glycemic control is paramount if they are to reach old age without significant morbidity. For those who develop diabetes later in life (>60 years) their phenotype appears to have provided them with some level of protection, but modest lifestyle changes and appropriate clinical management can assure their quality of life in later years. A range of evidence-based interventions including high-intensity interval training (HIIT), the dietary approaches to stop hypertension (DASH), smoking cessation, moderate alcohol consumption, bariatric surgery, and pharmacologic therapies targeting hyperglycemia, blood pressure, lipids and body weight are available to mitigate these risks (11–15). Given the significant burden of DKD among people with diabetes living in rural Australia, this perspective focuses on the latest evidence in screening, prevention, and treatment of DKD, with attention to emerging advances and rural healthcare implications.

### Chronic kidney disease in rural Australia

**Figure 2:**
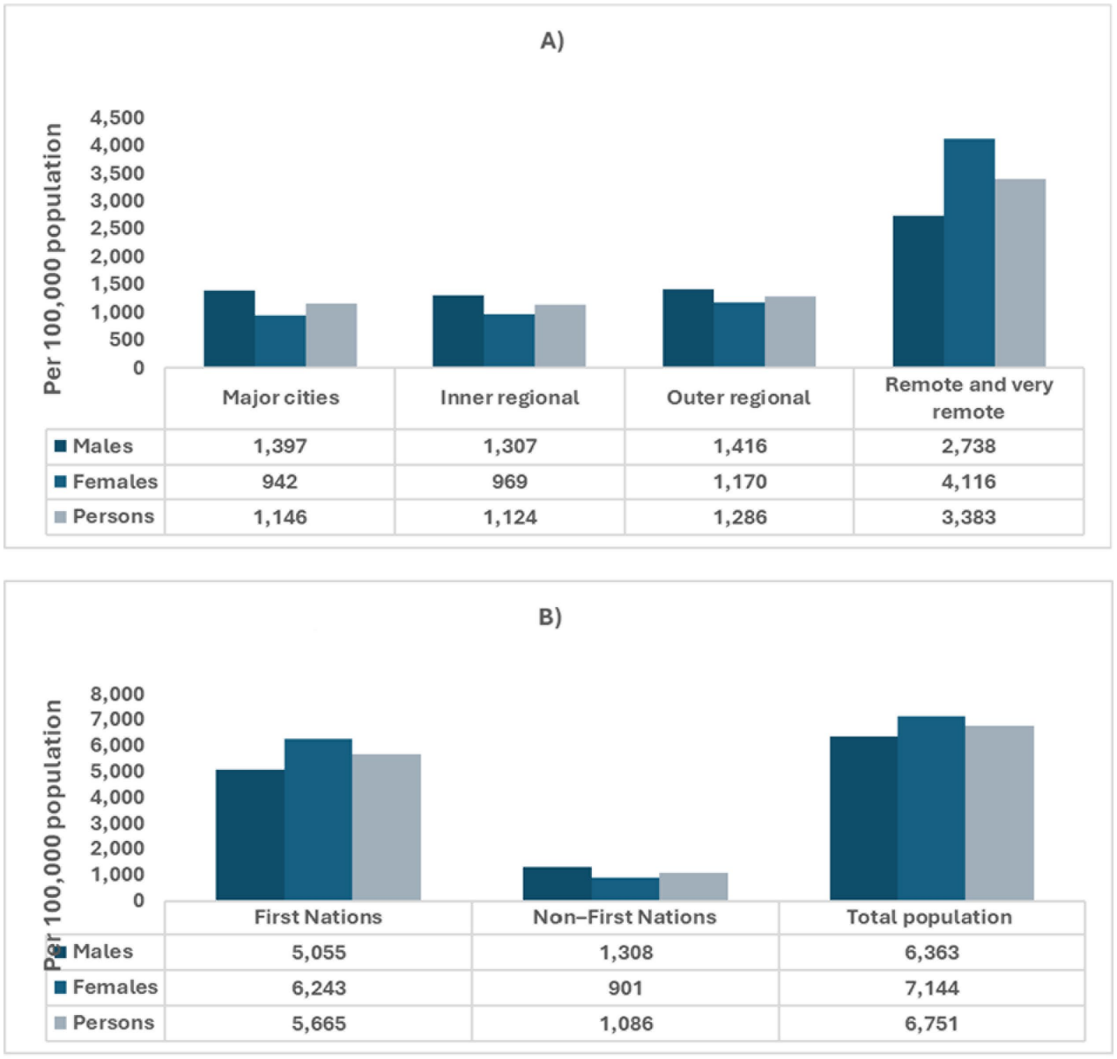
Age-adjusted hospitalizations for chronic kidney disease in Australia, categorized by sex, geographic remoteness, and Indigenous status. Data sources: chronic kidney disease: Australian facts, Data–Australian Institute of Health and Welfare (aihw.gov.au). **(A)** Age-standardized hospitalization for CKD by sex and remoteness. **(B)** Age-standardized hospitalization for CKD by Indigenous Status.

In Australia, chronic kidney disease (CKD) accounted for approximately 2 million hospitalizations, representing 18% of all hospital admissions in 2021–22 (16). Of these, dialysis was the leading cause, comprising 81% of CKD-related hospitalizations (1.7 million), with an estimated 598,000 involving individuals with diabetes. This indicates that around 5% of all hospitalizations in Australia were for dialysis in the treatment of diabetes-related kidney disease (17). Between 2000–01 and 2021–22, hospitalizations with CKD as the principal diagnosis (excluding dialysis) more than doubled, rising from 24,200 to 56,800, with a 57% increase in the age-standardized rate (16). Among individuals under 45, CKD hospitalization rates (as a principal or additional diagnosis, excluding dialysis) were 1.2 to 2.0 times higher for females compared to males (16) (Figure 2). CKD disproportionately affects Aboriginal and Torres Strait Islander peoples, individuals in lower socioeconomic areas, and those living in remote regions (4, 5, 7). In 2018, CKD contributed to 2.5% of the total disease burden among First Nations people, with a higher burden from mortality (73%) compared to morbidity (27%). The overall burden of CKD was 7.8 times greater for First Nations people than for non-Indigenous Australians (18). This disparity arises from a complex interplay of factors. First Nations populations face elevated rates of diabetes, hypertension, and obesity, all of which are significant risk factors for CKD. Limited access to healthcare services in remote regions further delays early diagnosis and effective management of the condition (4, 19). Socioeconomic challenges, including poverty, inadequate housing, and food insecurity, contribute to the amplification of these risks. Additionally, while genetic predispositions may influence the prevalence of CKD, further research is necessary to better understand their full impact (4, 19). In rural and remote areas, CKD remains a leading cause of hospitalizations (16). In 2021–22, there were 17,100 CKD hospitalizations in remote regions (3,383 per 100,000 people), with residents being three times more likely to be hospitalized for CKD than those in major cities (16) (Figure 2).

### Screening—spot urine UCAR, eGFR, and other diagnostic markers

According to the Royal Australian College of General Practitioners (RACGP) guidelines, adults aged 40 years and above should be assessed for their risk of developing type 2 diabetes every 3 years using the Australian Type 2 Diabetes Risk Assessment Tool (AUSDRISK) (14, 20). Designed for the Australian context, AUSDRISK has demonstrated greater sensitivity and accuracy in detecting undiagnosed diabetes compared to tools like FINDRISK, which was developed in Europe to predict 10-year diabetes risk using different lifestyle parameters, including dietary habits. AUSDRISK, by contrast, estimates 5-year diabetes risk (14, 20, 21). However, AUSDRISK is not recommended for use among Aboriginal and Torres Strait Islander peoples due to the significantly higher baseline prevalence of type 2 diabetes in these communities (14). The tool may underestimate their actual risk, and thus, the RACGP advises initiating annual blood glucose testing including fasting plasma glucose, random glucose, or HbA₁c from the age of 18 in First Nations adults. This direct approach allows for earlier diagnosis and management by bypassing risk scoring models that may not reflect the true disease burden in these populations (14). Opportunistic screening for other chronic conditions, such as cardiovascular risk, is also recommended from a similarly early age. The RACGP 2020 guidelines provide a step-by-step approach for screening and diagnosing type 2 diabetes in asymptomatic individuals. It outlines appropriate use of AUSDRISK, when to initiate blood tests, and how to interpret fasting glucose and HbA₁c results to determine the need for further assessment, lifestyle intervention, or diagnosis (14, 20).

In parallel, screening for diabetic kidney disease (DKD) in Australia is guided by RACGP and Diabetes Australia recommendations. Annual assessments using both urine albumin-to-creatinine ratio (UACR) and estimated glomerular filtration rate (eGFR) are advised for all individuals with type 2 diabetes, and for those with type 1 diabetes starting 5 years after diagnosis (14). In rural and remote settings, adherence to these recommendations is often compromised due to geographic isolation, limited access to nephrology services, and persistent workforce shortages (5). General practitioners (GPs) typically lead CKD screening, often supported by nurse practitioners and Aboriginal Health Practitioners, particularly in Aboriginal Community Controlled Health Organisations (ACCHOs) (7, 18). These systemic challenges highlight the need for more equitable kidney health strategies in rural Australia (4).

According to the American Diabetic Association (ADA) an individuals with type 2 diabetes should be screened for CKD at the time of diagnosis, while those with type 1 diabetes should begin screening 5 years after the initial diagnosis (9, 11, 22). The rationale for screening immediately at diagnosis in type 2 diabetes stems from the fact that many individuals may already have had the condition undiagnosed for years (9, 22). Recommended screening includes a random spot UACR test and an eGFR assessment (9, 22). Timed 24-h collections are burdensome, and immunoassays or dipstick tests for urinary albumin alone (without creatinine) are often not performed due to lowered sensitivity and specificity (9, 22). It is noteworthy that two of three samples for urine albumin-creatinine ratio (UACR) collected within 6 months should be abnormal, along with a reduced eGFR, for a diagnosis of CKD to be considered (9, 22). Diagnosis can be challenging because UACR values may be temporarily elevated by factors such as physical activity, infections, elevated body temperature, congestive heart failure (CHF), menstruation, and with extreme blood pressure or hyperglycemia (9, 22). Patients with CKD who have a normal eGFR (≥90 mL/min/1.73m^2^) and a normal UACR (<3 mg/mmol) require annual screening (9). Individuals with a reduced eGFR (30–60 mL/min/1.73m^2^) and an elevated (UACR >30 mg/mmol) should undergo screening twice annually (9). Patients with a significantly decreased eGFR (<30 mg/mmol) should be referred to a nephrology service for through evaluation and adjustment of treatment (9).

Recent advances in screening highlight neutrophil gelatinase-associated lipocalin (NGAL) as a promising biomarker (23). Studies have consistently shown that renal tubulointerstitial injury plays a key role in the progression of diabetic kidney disease (DKD) (23). NGAL, a tubular structural marker belonging to the lipocalin superfamily, rises markedly in the serum or urine of patients within hours following ischemia–reperfusion injury (23). A recent meta-analysis reported a pooled sensitivity of 0.79 and specificity of 0.87 for serum NGAL, based on seven studies with 1,238 participants (23). For urine NGAL, the pooled sensitivity was 0.85 and specificity was 0.74, from 10 studies involving 1,369 participants (23). Overall, NGAL shows promise for DKD classification and may also have a diagnostic value for normoalbuminuric kidney disease (23). Additionally, two other promising biomarkers for diagnosing DKD in type 2 diabetes are urinary kidney injury molecule 1 (uKIM-1) and Chitinase-3-like protein 1 (YKL-40) (24). KIM-1 is a type 1 epithelial transmembrane glycoprotein that is expressed in response to ischemic or toxic injury to the proximal kidney tubules, whereas YKL-40 is a 40 KDa chitin-binding glycoprotein used as an inflammatory marker and an indicator of endothelial dysfunction in both type 1 and type 2 diabetics, with levels increasing alongside albuminuria (24). A meta-analysis of 14 studies found that the AUC of uKIM-1 and YKL-40 for T2DM patients with normoalbuminuria was 0.85 and 0.91, suggesting their potential role in diagnosing diabetic kidney disease (24).

### Prevention—lifestyle changes and ongoing management

Both the Australian Diabetic Society and ADA recommended several specific evidence-based preventive measures to lower the risk and time of onset of microvascular/macrovascular complications. Some of these strategies include: maintaining glycated haemoglobin (HbA1c) under 7%; a blood pressure under 130/80 mmHg; achieving cholesterol targets of <4.0 mmol/L for total cholesterol, <2.0 mmol/L for low-density lipoprotein (LDL-C), and triglycerides, ≥1 mmol/L for high-density lipoprotein (HLD-C); maintaining a body mass index (BMI) within the normal range (e.g., 18.5–24.9 kg/m^2^); following a DASH or Mediterranean diet that is low in carbohydrates, sodium, and fat, but rich in dietary fibre and whole grains; consuming alcohol in moderation (e.g., ≤ 1–2 drinks per day); smoking cessation; engaging in minimum 150 min of aerobic exercise per week along with 2–3 sessions of resistance training (≥ 60 min per week); and ensuring at least 7–8 h of sleep per night (9, 12, 13, 22, 25–28). Additional lifestyle measures that have recently been shown to be helpful include: cardio workouts for younger diabetics that includes a component of HIIT (e.g., at 60–70% maximum heart rate) and for older patients, yoga; and dietary supplements including Zinc (20 mg/day) and Inulin-type fructans (ITF) (<20 g/day) (29–32). ITF, which consists of inulin, fructooligosaccharides, and galactooligosaccharides, is a linear fructan linked by *β* (2–1) bonds and is widely recognized as a form of prebiotic (31). A recent meta-analysis of 33 clinical trials demonstrated that ITF supplementation significantly reduced fasting blood glucose, HbA1c, and fasting insulin levels in individuals with prediabetes and T2DM (31).

In order to meet these challenging targets there must be Shared Decision Making on Creation of a Management Plan and an agreement and willingness on behalf of the patient (12, 33). SMART goals should be patient-centered and Specific, Measurable, Attainable, Relevant, and Time-Bound (12, 33). Initially patients will form such a plan with their primary-care physician but if their health deteriorates over time the patient may need to be referred to a Diabetic Specialty Center for medication adjustment and continued patient-centered care by a multi-disciplinary team (e.g., Diabetic Endocrinologist, Nephrologist, Dietitian, Psychologist etc.) (12, 33). Typically a patient will be seen by their general practitioner every 3–6 months in order to assess emotional well-being, medication adherence, weight, HbA1C status, blood pressure, lipids, eGFR, and self-monitoring blood glucose (12, 33). Patients will see the Diabetic Specialty Center annually (or more frequently if required) for specific follow-up and care. For patients over 70 years of age the targets should be relaxed (e.g., HbA1c < 8%; walking 5 days per week for 20–30 min) (34). It is noteworthy that losing weight and keeping the weight off is a significant challenge which few can maintain given the body’s intense desire to return to previous (obese) physiological state (13). Intense exercise regimes are also difficult to maintain thus one must set realistic goals based on the patient’s age, health, and motivation (12, 33, 34).

### Treatment—SGLT2 inhibitors, GLP-1RAs, ACE inhibitors, ARBs, and more

In Australia, the Pharmaceutical Benefits Scheme (PBS) subsidizes a range of medications for managing DKD, including angiotensin-converting enzyme (ACE) inhibitors, angiotensin II receptor blockers (ARBs), sodium-glucose co-transporter-2 (SGLT2) inhibitors, and glucagon-like peptide-1 receptor agonists (GLP-1 RAs) (3, 35). Finerenone, a novel non-steroidal mineralocorticoid receptor antagonist that has demonstrated efficacy in slowing the progression of diabetic kidney disease, was listed on the Pharmaceutical Benefits Scheme (PBS) from July 1, 2023, for eligible individuals with chronic kidney disease linked to type 2 diabetes (3, 35). Although Australia’s healthcare system offers universal coverage, individuals living in rural and remote regions often encounter barriers such as reduced pharmacy availability and periodic medicine shortages, which can compromise treatment adherence (35). While GPs are the primary coordinators of care, specialist services including endocrinology and nephrology are less accessible outside metropolitan areas, making telehealth and visiting specialist clinics essential alternatives for continued care (8).

Every effort must be made to achieve the glycemic target (HbA1c < 7%) to slow the progression of CKD (9, 22). Metformin is considered as the first-line treatment for all individuals with type 2 diabetes, including those with CKD (9, 12, 22, 36, 37). Sodium-glucose cotransporter-2(SGLT2) inhibitors and Glucagon-like peptide-1 receptor agonists (GLP-1RAs) are regarded as excellent second-line agents for people with CKD (9, 22, 36, 37). By decreasing renal tubular glucose reabsorption, body weight, systemic blood pressure, intraglomerular pressure, and albuminuria, SGLT2 inhibitors thereby slow the decline in glomerular filtration rate (GFR) (9). Moreover, SGLT2 inhibitors (e.g., empagliflozin, canaglifozin) reduce oxidative stress and NLRP3 inflammation (9). GLP-1RAs (e.g., liraglutide, semaglutide) are recommended because they reduce cardiovascular diseases (CVD) risk, hypoglycemia and slow GFR loss (9, 36, 37). Notably, canaglifozin can be prescribed for patients with an eGFR as low as 30 mL/min/1.73 m^2^ (9). ACE inhibitors or ARBs are the preferred first-line therapy for managing blood pressure in patients with diabetes, hypertension, an eGFR <60 mL/min/1.73 m^2^, and a UAC ≥ 30 mg/mmol, due to their established benefits in preventing the progression of chronic kidney disease (CKD) (9).

Recent advancements in treatment focus on the use of renin-angiotensin system inhibitors to slow the progression of renal damage in diabetic patients, specially targeting the angiotensin-converting enzyme (ACE) pathway (38, 39). The ACE2/Ang-(1–7)/Mas receptor axis functions as the counter-regulatory pathway of the renin-angiotensin system (38, 39). Notably, recent animal experiments indicate that the addition of cyclic angiotensin-(1–7) to an ACE inhibitor enhances its effectiveness compared to using the ACE inhibitor alone. This combination therapy may offer significant benefits for diabetic patients who do not achieve adequate responses from standard treatment (38, 39). Additionally, piperazine ferulate (PF), a derivative of ferulic acid, has demonstrated improvements in renal blood flow mechanics and microcirculation (40). A recent meta-analysis encompassing 12 clinical trials evaluated the efficacy of PF combined with irbesartan, revealing a substantially higher total effective rate for the combined therapy [odds ratio (OR) = 4.95; 95% CI, 3.11e7.58; *p* < 0.0001] compared to irbesartan alone (40). Moreover, statistically significant decreases were seen in the fasting blood glucose, 2-h plasma glucose, serum creatinine, UCAR, urinary β_2_-microglobulin and blood urea nitrogen (40). A large clinical trial is recommended to verify these impressive findings.

### Way forward

In Australia diabetes care is primarily coordinated through GPs working in outpatient, community-based practices (14). According to national guidelines, GPs play a central role in screening, diagnosis, and long-term management of DKD, often supported by practice nurses, credentialled diabetes educators, and allied health professionals such as dietitians and podiatrists (14, 41). Referral to diabetes specialists, including endocrinologists and nephrologists, is recommended for patients with advanced or complex disease, typically coordinated via outpatient hospital clinics (14). In metropolitan regions, multidisciplinary diabetes clinics are more accessible; however, in rural and remote settings, access to specialist services is limited due to geographic isolation, workforce shortages, and long waiting times (4). Telehealth and visiting outreach clinics have been implemented to help mitigate these disparities, though service gaps remain (7, 17). Workforce distribution data from AIHW indicate that access to diabetes nurse educators and renal dietitians is significantly lower in rural areas compared to major cities, contributing to delayed diagnoses and reduced continuity of care (7, 14). Strengthening integrated, team-based care and expanding rural diabetes infrastructure is critical to ensuring timely, equitable management across regions (7, 14).

Building on this understanding of the healthcare delivery framework, it is critical to address the persistent burden and management gaps in DKD across rural Australia. Despite recent progress in the treatment and management of diabetic kidney disease, morbidity and mortality rates associated with DKD persistently increase in rural Australia. This trend positions DKD as a leading cause of CKD, contributing significantly to the number of patients requiring dialysis (16, 17, 42–44). Implementing a comprehensive approach to improve health outcomes for rural Australians to reduce healthcare burden of CKD is crucial. Such approach should emphasize early diagnosis through enhanced screening programs, improve self-management, home-based care, and community-supported care through primary healthcare, all of which can slow CKD progression and improve quality of life (16, 17, 42–44).

Self-management plays a central role in preventing and managing CKD. Key elements include lifestyle modifications such as dietary changes, regular physical activity, smoking cessation, achieving optimal glycaemic control, managing blood pressure, controlling lipid levels, and using reno-protective pharmacotherapy (9, 12, 13, 22, 34, 44). Despite these interventions, many CKD cases are diagnosed at an advanced stage, often due to limited healthcare access and low community awareness of early signs and risk factors (44–46). To address this, robust screening strategies should be implemented to detect DKD and undiagnosed type 2 diabetes at earlier stage (43, 44). Community health clinics, mobile outreach services, and partnerships with local organizations increase healthcare reach in underserved areas (45, 47–49). Collaboration between primary healthcare providers, diabetes specialists, and renal care teams is essential for coordinated multidisciplinary care (43, 44). Establishing diabetes clinics in rural regional centres could serve as education and monitoring hubs, while also delivering specialized clinical services (42, 43, 45, 47–50). The use of digital health tools and telemedicine can further bridge the access gap, expanding reach to remote communities (42, 43, 45, 47–50). Additionally, empowering patients and families to manage CKD at home and within their communities is critical strategy. Community health workers are pivotal in supporting adherence, conducting home visits, and assisting in care coordination. Advocacy for health system reforms, including increased investment in rural diabetes infrastructure and workforce expansion particularly the recruitment of diabetes specialists to regional centres will be key to addressing persistent health disparities (42–45, 47–50).

A crucial step forward is implementing guideline-based strategies tailored to rural and Indigenous populations. This includes adopting the RACGP’s screening recommendations such as annual diabetes testing from age 18 for First Nations people and ensuring culturally safe, community-led management. Strengthening partnerships with Aboriginal Community Controlled Health Services and First Nations health organizations will be essential to improve prevention, early detection, and management of diabetic kidney disease in these high-risk communities.

## Data Availability

The original contributions presented in the study are included in the article/supplementary material, further inquiries can be directed to the corresponding author.
